# Massive hemothorax due to inferior phrenic artery injury after blunt trauma

**DOI:** 10.1186/s13017-015-0052-3

**Published:** 2015-11-24

**Authors:** Makoto Aoki, Kei Shibuya, Minoru Kaneko, Ayana Koizumi, Masato Murata, Jun Nakajima, Shuichi Hagiwara, Masahiko Kanbe, Yoshinori Koyama, Yoshito Tsushima, Kiyohiro Oshima

**Affiliations:** Department of Emergency Medicine, Gunma University Graduate School of Medicine, Maebashi, Gunma Japan; Department of Diagnostic and Interventional Radiology, Gunma University Graduate School of Medicine, Maebashi, Gunma Japan

**Keywords:** Inferior phrenic artery, Blunt trauma, Transcatheter arterial embolization, N-butyl cyanoacrylate

## Abstract

Injury to the inferior phrenic artery after blunt trauma is an extremely rare event, and it may occur under unanticipated conditions. This case report describes an injury to the left inferior phrenic artery caused by blunt trauma, which was complicated by massive hemothorax, and treated with transcatheter arterial embolization (TAE).

An 81 year-old female hit by a car while walking at the traffic intersection was transferred to the emergency department, computed tomography scanning revealed active extravasations of the contrast medium within the retrocrural space and from branches of the internal iliac artery. The patient underwent repeated angiography, and active extravasation of contrast medium was observed between the retrocrural space and the right pleural space originating from the left inferior phrenic artery. The injured left inferior phrenic artery was successfully embolized with N-butyl cyanoacrylate, resulting in stabilization of the patient’s clinical condition.

Inferior phrenic artery injury should be recognized as a rare phenomenon and causative factor for hemothorax. TAE represents a safe and effective treatment for this complication and obviates the need for a thoracotomy.

## Background

Injury to the inferior phrenic artery after blunt trauma is an extremely rare event, and it may occur under unanticipated conditions. In the present case, blunt trauma led to left inferior phrenic artery injury associated with massive hemothorax, which was treated with TAE alone. To the author’s knowledge, this is the first report of massive hemothorax due to inferior phrenic artery injury treated definitively by TAE. Furthermore, previous cases of inferior phrenic artery injury after blunt trauma are reviewed.

## Review

### Case presentation

Following a collision with a car while walking at the traffic intersection, an 81 year-old female was transferred to the emergency department by helicopter. The patient had medication for hypertension and wasn’t on antiplatelet or anticoagulant medications. On hospital arrival (50 min after injury) the patient was alert, with a systolic/diastolic blood pressure (SBP/DBP) of 126/86 mmHg, a heart rate of 110 beats/min. Physical examination revealed tenderness in the pelvic region and contusion in the left knee joint. Initial laboratory studies revealed the following values; hemoglobin, 12.0 g/dl; white blood cell (WBC) count, 11,500/μl; platelet count, 16.9 × 10^4^/μl; creatinine (Cr), 0.45 mg/dl; prothrombin time international ratio, 1.00; activated partial thromboplastin time, 26.5 s; Arterial blood gas analysis measured on arrival revealed the following values; pH, 7.430; PCO_2_, 30.6 mmHg; PO_2_, 69.0 mmHg; HCO_3_^−^, 21.8 mmol/l; base excess, −3.6 mmol/l; lactate, 2.4 mmol/l.

**Fig. 1:**
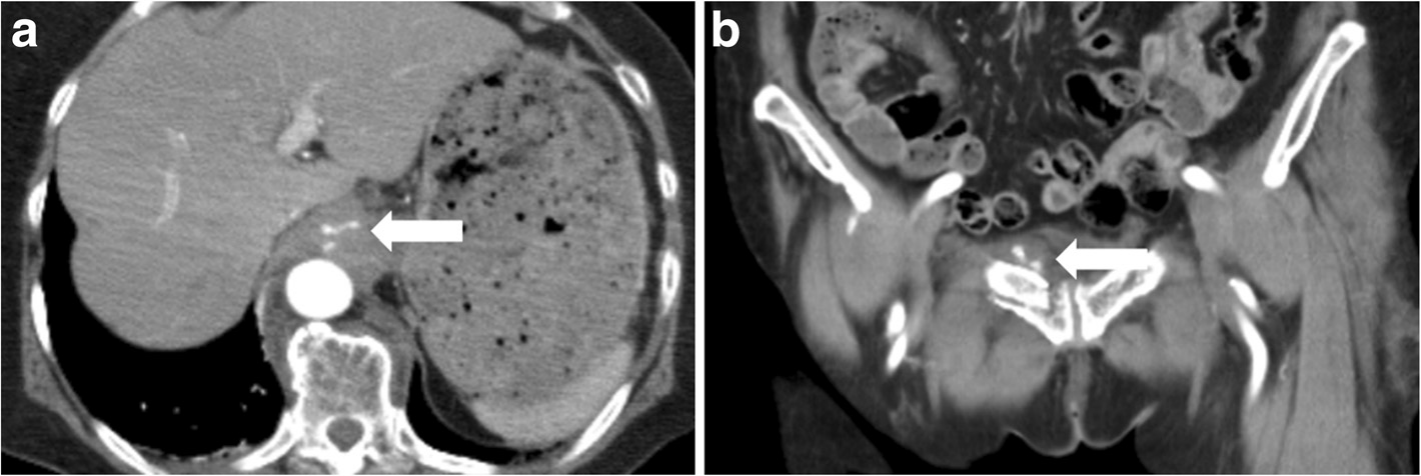
**a** Enhanced CT revealed contrast material extravasation within the retrocrural space on arterial phase (arrow). **b** Enhanced CT revealed contrast material extravasation above the pubic bone fractures on arterial phase (arrow)

**Fig. 2:**
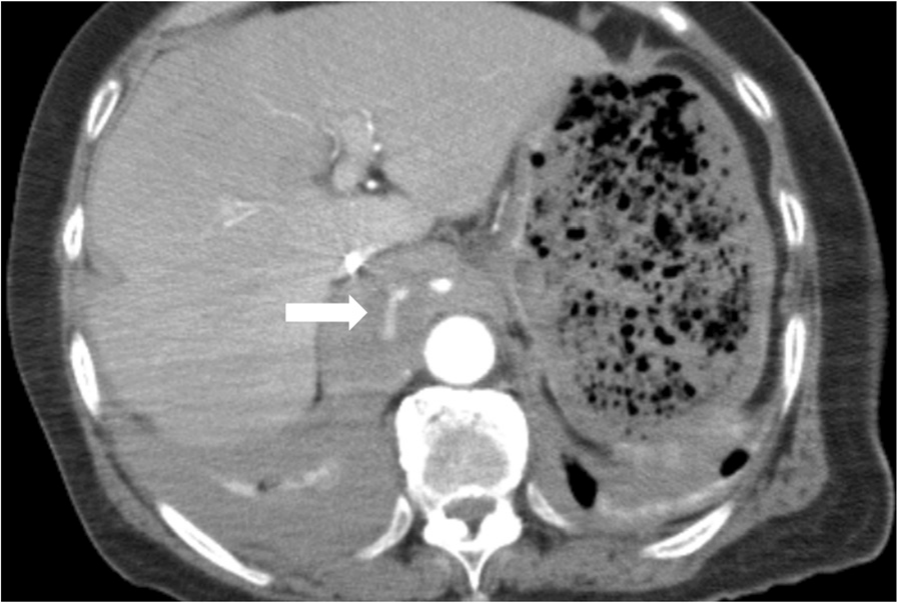
Enhanced CT showed the extension of the hematoma from within the retrocrural space to the right pleural space, and the extravasation of the retrocrural space (arrow)

**Fig. 3:**
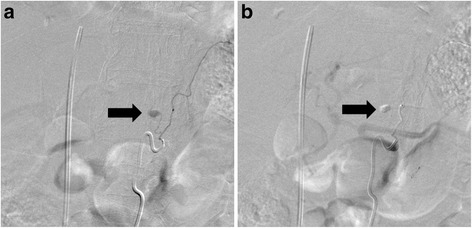
**a** Digital subtraction angiography of the left inferior phrenic artery angiography demonstrated contrast material extravasation (arrow). **b** After transcatheter arterial embolization, N-Butyl Cyanoacrylate (NBCA) and Lipiodol were detected (arrow)

Computed tomography (CT) scanning with contrast medium (80 min after injury) demonstrated that active extravasations were detected in the retrocrural space (Fig. 1a) and from branches of the internal iliac artery (Fig. 1b) with fractures to the pubic and ischial bones. In addition, No other injuries were observed in the abdominal solid organs, and obvious lung injury, rib fracture and diaphragm rupture were not found. In primary management intravenous lines were secured and a rapid infusion with normal saline was started, however, approximately 60 min after arrival, the patient’s vital signs became unstable (98/58 mmHg and 111 beats/min for SBP/DBP and heart rate, respectively). Immediately blood transfusion was started. To control the continuous bleeding from the pelvic fractures, angiography was performed. The right and left iliac arteries were cannulated with a 5-Fr cobra catheter (Medikit Co. Ltd., Tokyo, Japan). Digital subtraction angiography (DSA) of the internal iliac artery did not demonstrate obvious bleeding from both the internal and the external iliac artery, however, the patient situation was impending and embolization of the bilateral internal iliac arteries was empirically performed with gelatin sponge particles (Serescue; Nippon Kayaku Co. Ltd., Tokyo, Japan) based on the CT scans performed at arrival. CT scans performed during angiography demonstrated that the hematoma extended into the retrocrural space. Injury of the aorta and aortic branches was suspected. Each branch of aorta, comprising the left gastric artery, the celiac artery (including the left inferior phrenic artery) and superior mesenteric artery, was cannulated with a 5-Fr shepherd’s hook catheter (Terumo Clinical Supply Co. Ltd., Gifu, Japan) and a 5-Fr Michelson catheter (Medikit Co. Ltd., Tokyo, Japan). However, obvious extravasation was not confirmed. The patient recovered from shock and was transferred to the intensive care unit (ICU), but shock occurred again at 3 h post-ICU admission. Enhanced CT scans were perfomed again and revealed the hematoma extending from within the retrocrural space to the right pleural space, and extravasation within the retrocrural space (Fig. 2). Therefore, angiography was immediately repeated. The left inferior phrenic artery was cannulated with a 5-Fr Michelson catheter, and DSA of the left inferior phrenic artery showed extravasation (Fig. 3a). NBCA was mixed with iodized oil (Lipiodol; Andre Guerbet, Aulnay-sous-Bois, France) at a ratio of 1:3, and the mixture was injected. Post-embolization angiography was performed to confirm the absence of extravasation, and completion of TAE (Fig. 3b). Massive hemothorax in the right pleural space was demonstrated on post-embolization chest CT. A thoracostomy tube was inserted, and approximately 1000 ml of bloody fluid was collected. After TAE, the increase of hemothorax was not confirmed. The patient needed 14 units of red blood cells and 8 units of fresh frozen plasma within 24 h from injury. The post-treatment course was uneventful and clinical symptoms revealing a diaphragmatic injury were not seen. The patient was enrolled in orthopediac department for the operation of left knee joint on the 17^th^ day and transferred to another hospital for rehabilitation without complications on the 39^th^ day.

## Discussion

This was an unusual case of hemothorax because it was not accompanied by damage to the thoracic and abdominal organs. Only one case was previously reported in which hemothorax was caused by inferior phrenic artery injury, without multiple organ injury [1], however, this is the first report of an injured left inferior phrenic artery injury resulting in contralateral right hemothorax. Data from the present case suggest that damage to the left inferior phrenic artery injury led to a hemorrhage in mediastinum, which subsequently ruptured into the right pleural region.

The inferior phrenic artery originates between the middle of the second lumbar vertebrae and the twelfth thoracic vertebrae [2]. The right and left inferior phrenic arteries arise from the ascending (anterior), descending (posterior), superior suprarenal, and middle suprarenal branches. The ascending branch of the left inferior phrenic artery divides into the esophageal and accessory splenic branches [3]. According to various branches of the inferior phrenic artery, the injury of inferior phrenic artery has the potential to cause multiple clinical conditions. To date only four cases of inferior phrenic artery injury (excluding this report) have been reported, and each case was related to a different condition (Table 1) [1, 4–6]. TAE was selected as the treatment in four of the five reported cases, but different embolic materials were used. In the present case, TAE was performed for hemostasis, and NBCA was selected as the embolic material because the patient exhibited coagulopathy induced by severe trauma, with vital signs indicating shock. NBCA is considered to be the most appropriate embolic material for cases with hemorrhagic diathesis because it does not depend on the coagulation process for its therapeutic effect [7]. In contrast to Ogawa et al., we succeeded in complete treatment by TAE with NBCA [1]. This is the first report concerning an inferior phrenic artery injury complicated with massive hemothorax, and treated only by TAE using NBCA.

**Table 1:** The characteristics of the reported cases of inferior phrenic artery injury due to blunt trauma

Author	N	Clinical presentation	Diaphragmatic injury	Subsequent Treatment	Embolic material of TAE
Blaise	1	Pericardial tamponade	None	TAE	Polyvinyl alcohol particles
Mizobata	2	Intraperitoneal hemorrhage and Subcapsular hematoma	None	TAE	NS
Lee	1	Intrapetironeal hemorrhage	Grade V	Laparotomy	
Ogawa	1	Hemothorax	None	TAE and thoracotomy	Coil embolization
Aoki (present)	1	Hemothorax	None	TAE	NBCA

*N* number of patients, *TAE* transcatheter arterial embolization, *NS* not shown, *NBCA* N-butyl cyanoacrylate

Lee et al. described a patient with inferior phrenic artery injury accompanied by diaphragmatic injury, and laparotomy was selected [6]. Laparotomy is the best choice for single stage restoration. The present case was definitively treated by TAE and not accompanied by diaphragmatic injury, however, the combination of laparoscopy and thoracoscopy could be safe management and more useful for detecting the diaphragmatic injury [8].

In the other four cases, there were no concomitant injuries and complications. TAE may circumvent the need for thoracotomy or laparotomy, if the arterial injury is not associated with diaphragmatic rupture and stomach herniation into the left hemithorax [6]. TAE is commonly considered the most reliable and feasible therapeutic alternative to thoracotomy for control of intrathoracic arterial hemorrhages [9, 10] and is useful alternative treatment for a thoracotomy, which could be fatal in this 80+ year old patient. The authors propose that TAE represents the optimal strategy for management of inferior phrenic artery injury without diaphragmatic injury, and advances in microcatheter designs and embolic agents have contributed to the safety and effectiveness of TAE.

## Conclusions

In the summary we described the case of the left inferior phrenic artery injury who suffered blunt trauma. The findings suggest that hemothorax may be induced by inferior phrenic artery injury and this is very rare phenomenon. TAE can be a safe and effective treatment for the inferior phrenic artery bleeding and obviates the need for a thoracotomy.

### Consent statement

Written informed consent was obtained from the patient for publication of this Case report and any accompanying images. A copy of the written consent is available for review by the Editor-in-Chief of this journal.
